# Relatively high interest but limited active engagement in HIV cure research: Awareness, interest, and information-seeking among affected communities in the Netherlands

**DOI:** 10.1016/j.jve.2024.100570

**Published:** 2024-12-09

**Authors:** Maaike A.J. Noorman, John B.F. de Wit, Tamika A. Marcos, Sarah E. Stutterheim, Thijs Albers, Kai J. Jonas, Chantal den Daas

**Affiliations:** a Department of Interdisciplinary Social Science, Utrecht University, Utrecht, the Netherlands; b Department of Work and Social Psychology, Maastricht University, Maastricht, the Netherlands; c Department of Health Promotion and Care and Public Health Research Institute, Maastricht University, Maastricht, the Netherlands; d Hiv Vereniging, Amsterdam, the Netherlands; e Health Psychology Group Institute of Applied Health Sciences, University of Aberdeen, Aberdeen, United Kingdom

**Keywords:** HIV, HIV cure, Community engagement, People with HIV, GBMSM, Partners, MIPA

## Abstract

**Background:**

Community engagement is important for inclusive HIV cure development. This study evaluates current engagement in HIV cure research among affected communities in the Netherlands by analyzing awareness, interest, and information-seeking behavior. It also identifies participant characteristics and HIV-related illness perceptions linked to each engagement stage.

**Methods:**

A cross-sectional survey was conducted from July 2023 to March 2024, involving 499 people with HIV and 578 individuals without HIV, including partners and gay, bisexual, and other men who have sex with men. Multivariate regression analyses examined the relationships between participant characteristics, HIV-related illness perceptions, and three outcomes: awareness, interest, and information-seeking.

**Results:**

The mean awareness was 3.08 (SD = 0.99) interest was higher at 3.67 (SD = 0.85), while the information-seeking frequency was lower at 2.33 (SD = 0.97). Higher awareness was seen in older participants, non-cisgender men, and those with increased perceived control and comprehensibility of HIV. Interest in cure research was higher among people with HIV, those with a migration background, individuals with steady partner(s), and those experiencing greater HIV-related concerns, negative HIV-related emotions, and better HIV comprehension. Information-seeking frequency was greater among people with HIV, those with a bachelor's degree, individuals from a migration background, those with steady partner(s), and those perceiving more severe HIV-related symptoms, and heightened concerns and negative emotions about HIV.

**Conclusion:**

While moderate awareness exists, engagement remains passive with limited information-seeking; however, significant interest in a cure underscores the need for enhanced communication efforts to foster inclusive HIV cure development.

## 1 Introduction

Recently, there has been a surge in HIV cure research aimed at either eliminating the virus from the body (HIV eradication, clearance cure) or ART-free durable control of the virus (functional cure, HIV suppression).^1^ While much of this research focuses on biomedical, translational, and clinical sciences, the International AIDS Society (IAS) also emphasizes the importance of social engagement, particularly the engagement of affected communities, including people with HIV, their partners, and other key populations including GBMSM.^1^

The emphasis on community engagement reflects a broader trend within health psychology over the past 25 years, emphasizing social justice.^2^ Community engagement prioritizes strategies that draw from communities’ strengths and needs, fostering co-learning environments where individuals gain knowledge, skills and influence, empowering them to play an active role in research and decision-making.^2^ Recognized for its effectiveness in reducing health inequities and improving health outcomes, community engagement has become an important factor in health promotion and other health and social science disciplines.^2^^,^^3^ Evidence from the early HIV epidemic illustrates how people with HIV gained a “seat at the table” by educating themselves and blending lived experiences with scientific reasoning to advocate for their rights [^4^ pp284]. Successful community engagement has prompted health authorities, including the WHO, to revise guidelines, such as expanding early access to ART, and has been essential in HIV vaccine development by managing expectations and enhancing trial acceptance.^5^

Contrastingly, inadequate community engagement in pre-exposure prophylaxis (PrEP) research has been linked to the premature termination of several clinical trials in the early 2000s, resulting in delays in implementation.^6^^,^^7^ A meta-analysis further emphasized the importance of community involvement in PrEP adaptation, revealing that concerns about its efficacy and potential side effects were significant barriers to adoption. Additionally, it highlighted that awareness and knowledge are crucial enablers of PrEP uptake.^8^ This underscores how overlooked community perspectives can hinder not only the progress of development but also play an important role in the adaptation and implementation of biomedical advances.

Given these precedents, we contend that community engagement is also essential for the successful development and implementation of an HIV cure.^9^ Health behavior models can offer valuable insights into how affected communities can be effectively engaged in HIV cure research.^10^ The engagement process can be understood using stage models like the Precaution Adoption Process Model (PAPM), which outlines stages from being ‘unaware of the issue’ to ‘aware but not personally engaged,’ progressing to ‘engaged and deciding what to do,’ and moving toward ‘planning to act,’ ‘acting,’ and ‘maintaining.'^11^ Inspired by the first three stages, this study is one of the first to assess the current engagement with HIV cure research among affected communities in the Netherlands by evaluating their awareness, interest, and information-seeking behaviors.

While most studies focus on hypothetical scenarios, [e.g. Refs. 12, 13, 14, 15, 16, 17, 18] such as trial participation, few have explored actual community engagement in HIV cure research.^19^ To our knowledge, only one study from Hong Kong explicitly assessed awareness of ART-free durable control. That study found that less than half of people with HIV included in their study were aware of ART-free durable control, with treatment-experienced participants being more likely to be aware.^20^ Research from the United States and Brazil indicated that people had heard about HIV cure research, knew a cure was not yet available, or wanted to know more.^14^^,^^21^^,^^22^ Furthermore, our qualitative research in the Netherlands among people with HIV, their partners, and GBMSM without HIV revealed a general awareness of HIV cure research, though active engagement was uncommon.^23^^,^^24^

It also remains unclear who is aware, interested, and actively seeking information about HIV cure research, as engagement likely varies by age, gender, education, and context.^10^^,^^19^ Recent research also suggests that HIV-related illness perceptions – encompassing views on illness effects, control, symptoms, concerns, understanding, duration, and causes – may influence engagement.^19^^,^^23^, ^24^, ^25^ Therefore, in addition to evaluating current engagement, our study investigated the extent to which participant characteristics and HIV-related illness perceptions are associated with different stages of engagement. Lastly, we aimed to identify the specific characteristics and HIV-related illness perceptions that influence HIV cure research engagement among distinct communities, namely people with HIV and key populations without HIV.

## 2 Methods

### 2.1 Design

This cross-sectional online survey on social engagement in HIV cure research in the Netherlands was developed with input from community and professional advisory boards and offered in Dutch and English between July 2023 and March 2024. Following ethical guidelines, participants were not required to complete all questions, only those for informed consent and bot detection, and could exit the survey at any time. The study was approved by the Ethical Review Board of the Faculty of Social and Behavioral Science, Utrecht University (FETC20-373), and the Ethical Review Committee Psychology and Neuroscience, Maastricht University (188-11-02-2018_S21).

### 2.2 Participants and recruitment

Eligible participants included people with HIV and key populations without HIV (partners of people with HIV and GBMSM), aged 18 or older, residing in the Netherlands. Recruitment combined online (e.g., Instagram®, Grindr®) and offline strategies (magazines, community events, clinics), supported by stakeholders such as Hiv Vereniging (Dutch Association of 10.13039/100019769People with HIV), HIV care providers, Hello Gorgeous (part of national HIV alliance), Volle Maan (health communication agency), Amsterdam Municipal 10.13039/100018696Health Services (GGD), and Maastricht University's HIV Lab. The final sample comprised 1077 participants from affected communities who completed sections on HIV cure engagement, participant characteristics, and HIV-related illness perceptions, including 499 people with HIV and 578 key populations (533 GBMSM and 45 partners).

### 2.3 Measures

#### 2.3.1 Participant characteristics

Participant characteristics included age, gender (cis-gender men = assigned male at birth and identify as men; cis-gender women = assigned female at birth and identify as women; other), sexual orientation (gay/lesbian; heterosexual; bisexual; other), educational attainment (with or without bachelor's degree), migration background (no migration background = born in the Netherlands with both parents also born in the Netherlands; first-generation migration background = not born in the Netherlands; second-generation migration background = born in the Netherlands with one or both parents born abroad), and steady relationship status (with or without committed partner [s]).

For people with HIV, additional data included the year of diagnosis, disclosure status (someone; no one), and current ART use (yes; no). We measured the frequency of ART-related side effects and missed doses over the past month on a 5-point scale (1 = never, 5 = very often). Other HIV-related characteristics included whether people with HIV had switched or stopped their ART regimen (yes; no), latest viral load (undetectable; detectable; unknown), recent CD4 T-cell count (<500; >500; unknown), AIDS diagnosis history (yes; no; unknown), and prior participation in HIV-related medical research (yes; no). For key populations, we gathered data on PrEP use (yes; no) and HIV testing frequency (every 3 months; 6 months; every year; less than once a year; never).

#### 2.3.2 HIV-related illness perceptions

HIV-related illness perceptions were measured using a modified version of the Brief Illness Perception Questionnaire (Brief IPQ),^26^ adapted for people with HIV and key populations (Table S1). This scale evaluates seven dimensions: perceived consequences, perceived personal control, perceived treatment control, perceived identity, experienced concerns, experienced emotions, and perceived comprehensibility. The duration dimension from the Brief IPQ was excluded due to HIV's chronic nature. Participants responded on a 5-point scale (1 = not at all, 5 = very much). Higher scores for consequences, identity, concerns, and emotions indicated more threatening perceptions, while higher scores for control and comprehensibility reflected less threatening perceptions.

A principal factor analysis revealed two factors for people with HIV and three for key populations (Table S1). For both groups, the primary factor centered on the perceived impact of HIV, including dimensions such as consequences, concerns, identity, and emotions. Notably, the identity dimension was integrated into this factor for people with HIV only. Internal consistency for this factor was strong (people with HIV: Cronbach's α = 0.82; key populations: Cronbach's α = 0.79). The second factor, which captured perceived control and comprehensibility, demonstrated low internal consistency (people with HIV: Cronbach's α = 0.41; key populations: Cronbach's α = 0.45). A third factor, identified only for key populations, focused solely on perceived identity. Due to the divergent factor structures between people with HIV and key populations and the unsatisfactory Cronbach's alpha for the second factor, all items were analyzed individually.

#### 2.3.3 HIV cure engagement

Guided by the first three stages of the PAPM,^11^ we measured HIV cure engagement across three dimensions: awareness, interest, and information frequency. Awareness and interest were rated on a 5-point scale (1 = not at all, 5 = very much), while information-seeking frequency was reported on a 5-point scale (1 = never, 5 = often).

### 2.4 Data analysis

We conducted descriptive analyses to describe participant characteristics, HIV-related illness perceptions, and levels of engagement. Differences between people with HIV and key populations were examined using chi-square and ANOVA tests. Pearson correlation coefficients were calculated to explore relationships between awareness, interest, and information-seeking frequency for all affected communities and separately for people with HIV and key populations.

To evaluate how participant characteristics and HIV-related illness perceptions influence awareness, interest, and information-seeking frequency, we conducted three multivariable linear regression analyses. Bivariate linear regressions identified correlations among participant characteristics, HIV-related illness perceptions, and the three outcomes. To ensure inclusivity, all variables with a p < 0.10 from these analyses were incorporated into a standard multiple regression model. This process was repeated for people with HIV and key populations separately. Variables having p < 0.05 were deemed statistically significant. Since participants were not required to answer all questions (except for informed consent and bot detection), missing data were handled through listwise deletion. All analyses were conducted using SPSS version 29.0.0.0.

## 3 Results

### 3.1 Participant characteristics

The mean age was 47 years (*SD* = 14.14), with people with HIV significantly older (*M* = 54,*SD* = 12.51) than key populations (*M* = 42,*SD* = 13.34) (Table 1). Most participants identified as cisgender men (91.8 %), but people with HIV had a higher proportion of non-cisgender men (13.0 %) compared to key populations (4.0 %). Approximately 64.0 % held a bachelor's degree, with key populations having a higher rate (69.9 %) than people with HIV (57.1 %). About 25 % reported a migration background, with similar rates across both groups. Roughly 80 % identified as gay or lesbian, with a higher proportion among key populations (84.4 %) than people with HIV (78.0 %). Approximately half reported having one or more steady partners, with no significant differences between groups.

**Table 1:** Participant characteristics and HIV-related illness perceptions across people with HIV and key populations without HIV.

	All affected communities (N = 1077)		People with HIV (N = 499)		Key populations without HIV (N = 578)		Chi-square or ANOVA results	
	N (%)	M(SD)	N (%)	M(SD)	N (%)	M(SD)	χ^2^ (df) or F (df), η^2^ₚ	p-value
**Participant characteristics**								
Age (in years)		47.45 (14.14)		53.54 (12.51)		42.18 (13.34)	F (1, 1075) = 205.77, η^2^ₚ = 0.161	<0.001
Gender							χ2 (2) = 51.96	<0.001
Cis men	989 (91.8)		434 (87.0)		555 (96.0)			
Cis women	67 (6.2)		59 (11.8)		8 (1.4)			
Other	21 (1.9)		6 (1.2)		15 (2.6)			
Bachelor's degree							χ2 (1) = 18.65	<0.001
No	385 (35.7)		212 (42.5)		173 (29.9)			
Yes	689 (64.0)		285 (57.1)		404 (69.9)			
Migration background								0.284
No	799 (74.2)		380 (76.2)		419 (72.5)			
First generation	106 (9.8)		42 (8.4)		64 (11.1)			
Second generation	171 (15.9)		77 (15.4)		94 (16.3)			
Sexual identity							χ2 (3) = 70.53	<0.001
Gay/Lesbian	877 (81.4)		389 (78.0)		488 (84.4)			
Heterosexual	81 (7.5)		72 (14.4)		9 (1.6)			
Bisexual	79 (7.3)		24 (4.8)		55 (9.5)			
Other	40 (3.7)		14 (2.8)		26 (4.5)			
Steady partner(s)							χ2 (1) = 6.11	0.013
No	486 (45.1)		214 (42.9)		292 (50.5)			
Yes	519 (52.7)		283 (56.7)		285 (49.3)			
**HIV-related illness perceptions**								
Consequence		2.84 (0.99)		2.73 (1.00)		2.95 (0.96)	F (1, 1073) = 11.28, η^2^ₚ = 0.011	<0.001
Personal control		3.62 (0.91)		3.51 (1.02)		3.73 (0.78)	F (1, 1039) = 16.16, η^2^ₚ = 0.015	<0.001
Identity		2.67 (0.99)		2.22 (0.97)		3.08 (0.80)	F (1, 1034) = 247.64, η^2^ₚ = 0.193	<0.001
Treatment control		4.26 (0.82)		4.54 (0.69)		4.02 (0.84)	F (1, 1066) = 116.68, η^2^ₚ = 0.099	<0.001
Concern		2.58 (1.00)		2.23 (0.96)		2.88 (0.94)	F (1, 1072) = 123.70, η^2^ₚ = 0.104	<0.001
Emotions		2.58 (1.07)		2.37 (1.04)		2.77 (1.07)	F (1, 1056) = 39.14, η^2^ₚ = 0.036	<0.001
Comprehensibility		3.29 (0.73)		3.56 (0.67)		3.06 (0.70)	F (1, 1063) = 130.13, η^2^ₚ = 0.109	<0.001

*Note.* Since participants were not required to answer all questions (except for informed consent and bot detection) degrees of freedom may differ for across variables.

Most people with HIV were diagnosed between 1996 and 2020 (82.0 %), with 8.1 % diagnosed after 2020 and 9.9 % before 1996. Nearly all people with HIV (99.4 %) were on ART and demonstrated high adherence (*M* = 4.58,*SD* = 0.73), with 98.8 % achieving an undetectable viral load. Additionally, 94.4 % had disclosed their HIV status to someone. Among key populations, 62.9 % did not take PrEP, and most tested every 3 or 6 months (55.8 %).

### 3.2 HIV-related illness perceptions

HIV-related illness perceptions differed significantly between people with HIV and key populations (Table 1). Participants had a mean consequence score of 2.84 (*SD* = 0.99), with people with HIV perceiving consequences as less threatening (*M* = 2.73,*SD* = 1.00) compared to key populations (*M* = 2.95,*SD* = 0.96). The mean personal control score was 3.62 (*SD* = 0.91), with people with HIV reporting lower personal control (*M* = 3.51,*SD* = 1.02) than key populations (*M* = 3.73,*SD* = 0.78). For identity, the overall mean was 2.67 (*SD* = 0.99), with people with HIV exhibiting less threatening perceptions (*M* = 2.22,*SD* = 0.97) compared to key populations (*M* = 3.08,*SD* = 0.80). Treatment control scores averaged 4.26 (SD = 0.82), with people with HIV reporting more control (*M* = 4.54,*SD* = 0.69) than key populations (*M* = 4.02,*SD* = 0.84). Concern levels averaged 2.58 (*SD* = 1.00), with people with HIV showing less concern (*M* = 2.23,*SD* = 0.96) than key populations (*M* = 2.88,*SD* = 0.94). The mean score for emotions was 2.58 (*SD* = 1.07), with people with HIV reporting less intense emotions (*M* = 2.37,*SD* = 1.04) compared to key populations (*M* = 2.77,*SD* = 1.07). Lastly, comprehensibility averaged 3.29 (*SD* = 0.73), with people with HIV scoring higher (*M* = 3.56,*SD* = 0.67) than key populations (*M* = 3.06,*SD* = 0.70).

### 3.3 HIV cure research engagement

**Fig. 1:**
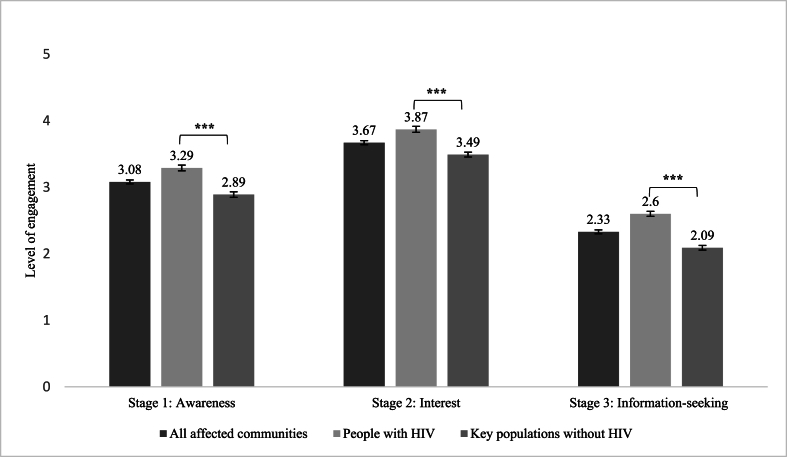
Mean and standard error for the level of HIV cure engagement.

Participants reported a mean awareness of 3.08 (*SD* = 0.99) and a higher mean interest of 3.67 (*SD* = 0.85), while the mean frequency of information-seeking was lower at 2.33 (*SD* = 0.97) (Fig. 1). People with HIV scored higher than key populations in awareness (*F* [1,1075] = 42.67,*η*^*2*^*ₚ* = 0.038, *p* < 0.001), interest (*F* [1,1074] = 76.76,*η*^*2*^*ₚ* = 0.067, *p* < 0.001), and information-seeking (*F* [1,1074] = 55.89,*η*^*2*^*ₚ* = 0.049, *p* < 0.001).

Table 2 shows a weak positive correlation between awareness and interest across all affected communities (*r* = 0.060,*p* < 0.05), which was not significant for people with HIV and key populations. In contrast, awareness had a strong correlation with information-seeking frequency across all communities (*r* = 0.500,*p* < 0.001), as well as for people with HIV (*r* = 0.433,*p* < 0.001) and key populations (r = 0.517,*p* < 0.001). Additionally, interest significantly correlated with information-seeking frequency across all communities (*r* = 0.321,*p* < 0.001), with slightly stronger correlations among people with HIV (*r* = 0.336,*p* < 0.001) than key populations (*r* = 0.228,*p* < 0.001).

**Table 2:** Pearson correlations for engagement variables.

	1			2			3		
	All affected communities	People with HIV	Key populations	All affected communities	People with HIV	Key populations	All affected communities	People with HIV	Key populations
1. Awareness	–	–	–						
2. Interest	0.060*	0.006	0.027	–	–	–			
3. Information-seeking frequency	0.500***	0.433***	0.517***	0.321***	0.336***	0.228***	–	–	–

p <.05 ∗∗p < .01 ∗∗∗p<0.001

### 3.4 Multivariable linear regression analyses

#### 3.4.1 Awareness

Awareness among affected communities was significantly higher in older participants (*B* = 0.007,*SE* = 0.002), non-cisgender men (*B* = 0.187,*SE* = 0.078), and those with greater perceived personal (*B* = 0.095,*SE* = 0.034) and treatment control (*B* = 0.078, *SE* = 0.038) over HIV, as well as higher perceived comprehensibility of HIV (*B* = 0.474,*SE* = 0.043) (Table 3).

**Table 3:** Bivariate and multivariate regression analysis of the level of HIV cure awareness, interest, and information seeking among all affected communities.

	Awareness		Interest		Information seeking	
	Bivariate Analyses	Multivariate analysis (N = 993)	Bivariate Analyses	Multivariate analysis (N = 993)	Bivariate Analyses	Multivariate analysis (N = 1012)
	Unstandardized Beta (SE)	Unstandardized Beta (SE)	Unstandardized Beta (SE)	Unstandardized Beta (SE)	Unstandardized Beta (SE)	Unstandardized Beta (SE)
**Participant characteristics**						
HIV-status *Ref.* People with HIV	−0.389 (0.059)∗∗∗	0.010 (0.077)	−0.378 (0.051)∗∗∗	**−0.432 (0.063)∗∗∗**	−0.502 (0.057)∗∗∗	**−0.357 (0.074)∗∗∗**
Age (in years)	0.012 (0.002)∗∗∗	**0.007 (0.002)∗∗**	−0.001 (0.002)		0.007 (0.002)∗∗∗	**0.005 (0.002)∗**
Gender *Ref.* cisgender male	0.189 (0.084)∗	**0.187 (0.078)∗**	0.150 (0.072)∗	0.096 (0.068)	0.242 (0.082)∗∗	0.014 (0.080)
Bachelor's degree *Ref.* No	0.064 (0.063)		−0.005 (0.054)		−0.124 (0.062)∗	**−0.157 (0.061)∗**
Migration background *Ref.* No	−0.011 (0.040)		0.147 (0.034)∗∗∗	**0.089 (0.034)∗∗**	0.170 (0.039)∗∗∗	**0.123 (0.039)∗∗**
Sexual identity: *Ref:* gay/lesbian	−0.044 (0.039)		−0.040 (0.034)		0.083 (0.038)∗	0.055 (0.038)
Partner(s): *Ref.* No	0.196 (0.060)∗∗	0.098 (0.058)	0.135 (0.052)∗∗	**0.124 (0.050)∗**	0.145 (0.059)∗	**0.118 (0.057)∗**
**HIV-related illness perceptions**						
Consequence	−0.081 (0.031)∗∗	−0.032 (0.038)	0.125 (0.026)∗∗∗	−0.008 (0.033)	0.087 (0.030)∗∗	−0.032 (0.037)
Personal control	0.164 (0.033)∗∗∗	**0.095 (0.034)∗∗**	−0.063 (0.029)∗	−0.002 (0.030)	0.018 (0.033)	
Identity	−0.143 (0.031)∗∗∗	−0.065 (0.034)	−0.044 (0.027)^+^	−0.022 (0.030)	−0.102 (0.030)∗∗∗	**−0.080 (0.034)∗**
Treatment control	0.267 (0.036)∗∗∗	**0.078 (0.038)∗**	0.094 (0.032)∗∗	0.033 (0.033)	0.137 (0.036)∗∗∗	0.009 (0.037)
Concern	−0.141 (0.030)∗∗∗	−0.008 (0.040)	0.139 (0.026)∗∗∗	**0.150 (0.035)∗∗∗**	0.110 (0.029)∗∗∗	**0.190 (0.040)∗∗∗**
Emotions	−0.081 (0.028)∗∗	0.033 (0.035)	0.154 (0.024)∗∗∗	**0.125 (0.031)∗∗∗**	0.102 (0.028)∗∗∗	**0.090 (0.035)∗**
Comprehensibility	0.544 (0.038)∗∗∗	**0.474 (0.043)∗∗∗**	0.192 (0.035)∗∗∗	**0.116 (0.038)∗∗**	0.331 (0.040)∗∗∗	**0.292 (0.043)∗∗∗**

p < 0.10^+^, p < 0.05∗, p < 0.01∗∗p < 0.001∗∗∗.

*Note.* Since participants were not required to answer all questions (except for informed consent and bot detection), missing data were handled through listwise deletion resulting in varying participant numbers per outcome.

**Bold** means statistically significant in the multivariate model.

Awareness R^2^ = 0.205; Interest R^2^ = 0.160; Interest R^2^ = 0.181.

For people with HIV, greater awareness correlated with higher perceived personal control (*B* = 0.157,*SE* = 0.044), increased HIV-related comprehensibility (*B* = 0.440,*SE* = 0.052), and better self-reported adherence (*B* = 0.149,*SE* = 0.054) (Table 4). Among key populations, greater awareness was associated with older age (*B* = 0.008,*SE* = 0.003), increased perceived comprehensibility (*B* = 0.440,*SE* = 0.049), and higher treatment control (*B* = 0.154,*SE* = 0.049). Key populations who tested more regularly (*B* = −0.051,*SE* = 0.022) and reported fewer concerns about HIV (*B* = −0.091,*SE* = 0.042) also showed greater awareness (Table 5).

**Table 4:** Bivariate and multivariate regression analysis of HIV cure awareness, interest and information seeking among people with HIV.

	Awareness		Interest		Information seeking	
	Bivariate Analyses	Multivariate analysis (N = 486)	Bivariate Analyses	Multivariate analysis (N = 474)	Bivariate Analyses	Multivariate analysis (N = 478)
	Unstandardized Beta (SE)	Unstandardized Beta (SE)	Unstandardized Beta (SE)	Unstandardized Beta (SE)	Unstandardized Beta (SE)	Unstandardized Beta (SE)
**Participant characteristics**						
Age (in years)	0.002 (0.003)		−0.007 (0.003)∗	0.001 (0.003)	−0.001 (0.004)	
Gender *Ref.* cisgender male	0.174 (0.110)		0.043 (0.096)		0.169 (0.117)	
Bachelor's degree *Ref.* No	0.009 (0.085)		−0.040 (0.075)		−0.073 (0.091)	
Migration background *Ref.* No	−0.081 (0.057)		0.154 (0.049)∗∗	0.077 (0.053)	0.151 (0.060)∗	0.096 (0.060)
Sexual identity: *Ref:* gay/lesbian	0.019 (0.061)		0.052 (0.053)		0.218 (0.064)∗∗∗	**0.190 (0.063)∗∗**
Partner(s): *Ref.* No	0.095 (0.085)		0.061 (0.075)		0.133 (0.091)	
Years of diagnosis	0.003 (0.005)		0.013 (0.004)∗∗	0.009 (0.005)	0.014 (0.005)∗∗	0.007 (0.005)
On treatment *Ref.* Yes	1.053 (0.543)		0.800 (0.475)		1.407 (0.577)∗	**1.079 (0.542)∗**
Side effects	−0.023 (0.037)		0.049 (0.032)		0.044 (0.040)	
Adherence	0.152 (0.057)∗∗	**0.149 (0.054)∗∗**	0.064 (0.050)		0.036 (0.061)	
Viral Load *Ref.* undetectable	−0.002 (0.316)		−0.014 (0.275)		−0.024 (0.337)	
CD4-count *Ref.* Less than 500 cells/mm^3^	−0.076 (0.066)		0.049 (0.058)		−0.090 (0.071)	
AIDS *Ref.* Yes	−0.042 (0.097)		0.073 (0.085)		0.135 (0.103)	
Disclosure *Ref.* To someone	−0.417 (0.182)∗	−0.241 (0.173)	0.022 (0.160)		−0.334 (0.194)	
Participation in Medical trial *Ref.* Yes	−0.222 (0.084)∗∗	−0.148 (0.080)	0.145 (0.073)∗	0.023 (0.076)	−0.034 (0.090)	
**HIV-related illness perceptions**						
Consequence	−0.056 (0.042)		0.181 (0.036)∗∗∗	0.095 (0.051)	0.109 (0.045)∗	−0.018 (0.59)
Personal control	−0.218 (0.052)∗	**0.157 (0.044)∗∗∗**	−0.021 (0.036)		0.073 (0.044)^+^	**0.096 (0.047)∗**
Identity	−0.088 (0.043)∗	−0.039 (0.049)	0.102 (0.038)∗∗	−0.023 (0.047)	0.062 (0.046)	
Treatment control	0.099 (0.040)∗∗∗	0.049 (0.062)	−0.078 (0.053)		−0.062 (0.065)	
Concern	−0.090 (0.044)∗	0.063 (0.053)	0.226 (0.037)∗∗∗	**0.102 (0.050)∗**	0.227 (0.046)∗∗∗	**0.244 (0.060)∗∗∗**
Emotions	−0.078 (0.040)^+^	−0.007 (0.049)	0.206 (0.034)∗∗∗	0.093 (0.050)	0.150 (0.043)∗∗∗	0.049 (0.057)
Comprehensibility	0.494 (0.059)∗∗∗	**0.440 (0.062)∗∗∗**	0.019 (0.055)		0.289 (0.065)∗∗∗	**0.336 (0.065)∗∗∗**

p < 0.10^+^, p < 0.05∗, p < 0.01∗∗p < 0.001∗∗∗.

*Note.* Since participants were not required to answer all questions (except for informed consent and bot detection), missing data were handled through listwise deletion resulting in varying participant numbers per outcome.

**Bold** means statistically significant in the multivariate model.

Awareness R^2^ = 0.174; Interest R^2^ = 0.111; Interest R^2^ = 0.153.

**Table 5:** Bivariate and multivariate regression analysis of HIV cure awareness, interest and information seeking among key populations without HIV.

	Awareness		Interest		Information seeking	
	Bivariate Analyses	Multivariate analysis (N = 534)	Bivariate Analyses	Multivariate analysis (N = 553)	Bivariate Analyses	Multivariate analysis (N = 526)
	Unstandardized Beta (SE)	Unstandardized Beta (SE)	Unstandardized Beta (SE)	Unstandardized Beta (SE)	Unstandardized Beta (SE)	Unstandardized Beta (SE)
**Participant characteristics**						
Age (in years)	0.011 (0.003)∗∗∗	**0.008 (0.003)∗**	−0.008 (0.003)∗∗	**−0.006 (0.003)∗**	0.000 (0.003)	
Gender *Ref.* cisgender male	0.090 (0.124)		0.156 (0.103)		0.172 (0.109)	
Bachelor's degree *Ref.* No	0.140 (0.091)		0.136 (0.075)^+^	0.033 (0.076)	−0.039 (0.080)	
Migration background *Ref.* No	0.060 (0.055)		0.155 (0.045)∗∗∗	0.087 (0.046)	0.205 (0.048)∗∗∗	**0.131 (0.049)∗∗**
Sexual identity: *Ref:* gay/lesbian	−0.078 (0.050)		−0.097 (0.042)∗	**−0.089 (0.041)∗**	0.006 (0.044)	
Partner(s): *Ref.* No	0.230 (0.083)∗∗	0.089 (0.080)	0.123 (0.069)^+^	**0.164 (0.069)∗**	0.086 (0.073)	
PrEP *Ref.* Yes	−0.350 (0.085)∗∗∗	−0.066 (0.104)	−0.262 (0.071)∗∗∗	**−0.178 (0.090)∗**	−0.237 (0.075)∗∗	−0.080 (0.096)
HIV testing *Ref.* every 3 months	−0.078 (0.018)∗∗∗	**−0.051 (0.022)∗**	−0.045 (0.015)∗∗	0.000 (0.019)	−0.060 (0.016)∗∗∗	−0.027 (0.020)
**HIV-related illness perceptions**						
Consequence	−0.067 (0.043)		0.115 (0.036)∗∗	**−0.102 (0.046)∗**	0.119 (0.038)∗∗	−0.023 (0.049)
Personal control	0.159 (0.054)∗∗	−0.015 (0.053)	−0.064 (0.045)		0.021 (0.048)	
Identity	−0.049 (0.054)		−0.017 (0.044)		−0.053 (0.047)	
Treatment control	0.248 (0.048)∗∗∗	**0.154 (0.049)∗∗**	0.084 (0.041)∗	0.038 (0.042)	0.112 (0.043)∗	0.081 (0.044)
Concern	−0.085 (0.044)^+^	**−0.091 (0.042)∗**	0.222 (0.036)∗∗∗	**0.178 (0.051)∗∗∗**	0.199 (0.038)∗∗∗	**0.120 (0.054)∗**
Emotions	−0.023 (0.040)		0.185 (0.032)∗∗∗	0.104 (0.040)∗∗	0.155 (0.034)∗∗∗	0.075 (0.042)
Comprehensibility	0.527 (0.56)∗∗∗	**0.440 (0.058)∗∗∗**	0.202 (0.049)∗∗∗	**0.132 (0.052)∗**	0.211 (0.052)∗∗∗	**0.131 (0.054)∗**

p < 0.10^+^, p < 0.05∗, p < 0.01∗∗p < 0.001∗∗∗.

*Note.* Since participants were not required to answer all questions (except for informed consent and bot detection), missing data were handled through listwise deletion resulting in varying participant numbers per outcome.

**Bold** means statistically significant in the multivariate model.

Awareness R^2^ = 0.196; Interest R^2^ = 0.142; Interest R^2^ = 0.099.

#### 3.4.2 Interest

Interest in HIV cure research among affected communities was significantly higher among people with HIV (*B* = −0.432,*SE* = 0.063), those with a migration background (*B* = 0.089,*SE* = 0.034), and individuals with steady partners (*B* = 0.124,*SE* = 0.050). Participants expressing greater concerns (*B* = 0.150,*SE* = 0.035), negative emotions about HIV (*B* = 0.125,*SE* = 0.031), and a higher comprehensibility of HIV (*B* = 0.116,*SE* = 0.038) also showed increased interest (Table 3).

Among people with HIV, only those with greater concerns (*B* = 0.102,*SE* = 0.050) demonstrated significantly higher interest (Table 4). In key populations, younger individuals (*B* = −0.006,*SE* = 0.003), gay or lesbian individuals (*B* = −0.089,*SE* = 0.041), those without partners (*B* = 0.164,*SE* = 0.069), and those on PrEP (*B* = −0.178,*SE* = 0.090) exhibited greater interest. Additionally, key populations who perceived less consequence from HIV (*B* = −0.102, *SE* = 0.051), had more concerns (*B* = 0.178,*SE* = 0.051), and higher HIV-related comprehensibility (*B* = 0.132,*SE* = 0.052) were more interested (Table 5).

#### 3.4.3 Information seeking

Among affected communities, information-seeking frequency was significantly higher among older (*B* = 0.005,*SE* = 0.002), people with HIV (*B* = −0.357,*SE* = 0.074), individuals without a bachelor's degree (*B* = −0.078,*SE* = 0.061), those with a migration background (*B* = 0.096,*SE* = 0.039), and those with one or more partners (*β* = 0.080,*SE* = 0.057). It was also associated with a stronger HIV identity (*B* = −0.082,*SE* = 0.034), greater concerns (*B* = 0.190,*SE* = 0.040), negative emotions (*B* = 0.090,*SE* = 0.035), and increased HIV comprehensibility (*B* = 0.292,*SE* = 0.043) (Table 3).

For people with HIV, information-seeking was notably higher among non-gay/lesbian participants (*B* = 0.190,*SE* = 0.063), those with more concerns (*B* = 0.244,*SE* = 0.060), higher perceived personal control (*B* = 0.096,*SE* = 0.047), and greater HIV comprehensibility (*B* = 0.336,*SE* = 0.065). Participants not on treatment (*B* = 1.079,*SE* = 0.542) were also more likely to seek information about HIV cure research (Table 4). In key populations, greater information-seeking was found among individuals with a migration background (*B* = 0.131,*SE* = 0.049), those expressing more concerns (*B* = 0.120,*SE* = 0.054), and those with higher perceived comprehensibility (*B* = 0.131,*SE* = 0.054) (Table 5).

## 4 Discussion

This research is one of the first to assess the non-hypothetical engagement of affected communities in HIV cure research by evaluating their awareness, interest, and information-seeking behavior, inspired by the early stages mapped out in the (PAPM).^11^ We also investigated which affected communities, participant characteristics, and HIV-related illness perceptions were associated with these three stages. Lastly, we examined if associated characteristics differed between the two groups of affected communities included in this research: people with HIV and key populations.

Our findings indicate that engagement with HIV cure research in affected communities is largely passive, characterized by low active information-seeking despite notable interest levels. Correlations between awareness and interest were weak across all communities and not significant among people with HIV and key populations, challenging the PAPM's assumption that greater awareness leads to higher interest.^10^^,^^11^ However, higher interest was associated with more frequent information-seeking, indicating that survey participation may have increased awareness and stimulated interest in HIV cure research. Alternatively, the lack of a clear definition of HIV cure research may have led to uncertainty or underconfidence in participants' self-reported awareness, consistent with previous research in the Netherlands that highlighted uncertainty between general awareness and detailed understanding.^24^^,^^27^ Future research should assess awareness of specific HIV cure developments for clearer insights.

People with HIV showed greater engagement across all stages compared to key populations, but HIV status did not significantly influence awareness. Instead, awareness was linked to individuals' perceived comprehensibility and control over HIV, which also influenced their interest and information-seeking frequency. This may be due to individuals who perceive higher comprehensibility and control over HIV having a stronger sense of agency and a desire to manage their health, prompting them to seek and retain more information about ongoing advancements in HIV cure research.^28^

Awareness of HIV cure research was also influenced by age and gender; however, older individuals and women did not show a corresponding increase in interest. This discrepancy may stem from their greater involvement in community settings,^2^^,^^29^ which enhances overall knowledge, including HIV cure awareness. For instance, the women's group 'Posidivas' has recently gained attention,^30^ and since 2022, the Hiv Vereniging has been chaired by a cisgender woman,^31^ fostering engagement and knowledge among women. Similarly, the ‘Long Term Survivors’, those diagnosed before 1996 and an active group within the community,^32^ likely keeps members informed. This aligns with research showing that HIV activists are often more willing to participate in cure trials.^15^ Further research should explore how community connectedness relates to HIV cure awareness and engagement.

Subgroup analyses indicated that ART adherence among people with HIV and frequent testing among key populations were linked to greater awareness. This suggests that individuals who manage their health,^28^ and engage regularly with healthcare professionals are more aware, a finding supported by studies highlighting the critical role of healthcare professionals in promoting HIV cure engagement.^20^^,^^33^, ^34^, ^35^

In contrast to awareness, both interest and information-seeking frequency were significantly higher among people with HIV. Individuals with greater concerns and negative emotions about HIV, a migration background, or steady relationships also demonstrated increased interest and information-seeking, suggesting that a heightened perceived need for an HIV cure drives these behaviors. People with HIV likely feel a stronger need for a cure due to its significant impact on their lives,^24^^,^^36^ as do those with heightened concerns and negative emotions.^24^ Individuals from migration backgrounds may face increased stigma,^37^ leading to poorer psychosocial and clinical outcomes,^38^^,^^39^ which heightens their perceived need for a cure.^24^ For those in steady relationships, a cure may be desired as it may alleviate transmission concerns.^23^^,^^36^ Future research should explore how perceived needs influence interest and information-seeking behavior.

Additionally, interest levels among key populations were influenced by younger age, identifying as gay, and PrEP use. Previous research suggests that the potential for an HIV cure to enhance sexual freedom, by eliminating transmission risk and reducing HIV-related anxieties, may drive this interest.^23^^,^^36^ Further investigation is needed to assess the extent to which the prospect of increased sexual freedom impacts interest in HIV cure research, including potential concerns about the risk of reinfection post-cure.

Lastly, our findings indicated that individuals without a bachelor's degree sought information about HIV cure research more frequently. Subgroup analyses revealed that people with HIV who are not on treatment and those who do not identify as gay also sought information more often. However, these groups did not demonstrate higher interest levels, suggesting that the available resources may not adequately meet their needs, prompting them to seek additional information.^40^ Further research is needed to identify community members facing challenges in accessing HIV cure information and to tailor resources accordingly.

Our study is among the first to evaluate non-hypothetical engagement in HIV cure research across diverse affected communities. However, several limitations should be noted. Although we highlighted the importance of HIV-related illness perceptions, our measurement tool showed inconsistent factor loadings and low internal consistency, likely due to high participant diversity and varied responses. For example, identity emerged as a distinct factor for key populations, rather than being part of the perceived impact factor, as it was for people with HIV. This may be because key populations have indirect experiences with HIV, leading to more varied and potentially unrealistic or threatening perceptions.^23^^,^^41^ Future studies should investigate how HIV-related illness perceptions form and the factors that shape them to refine the adapted Brief IPQ for both people with HIV and key populations.

Despite our sample's diversity, we may not have fully captured the range of experiences within the people with HIV and key populations in the Netherlands, particularly because partners of people with HIV were notably underrepresented. This limits our ability to draw specific conclusions about their perspectives, which likely differed from GBMSM.^24^ Future research should address this gap. Although we received input from advisory boards and used inclusive materials like information videos, the abstract nature of HIV cure research may have hindered recruitment, especially among individuals without bachelor's degrees. Future studies should consider assisting underserved communities in completing surveys to ensure more diverse participation. Additionally, while the Dutch context may resonate with experiences in other high-income countries, these findings may not be applicable in settings lacking access to ART or PrEP. Future research should explore these dynamics in diverse global contexts to enhance the broader applicability of the findings.

In conclusion, while moderate awareness exists—driven by knowledge, community ties, and healthcare interactions—engagement remains passive, with limited information-seeking. However, significant interest, fueled by the perceived need for a cure, is evident. To foster the active and meaningful engagement advocated by the IAS HIV cure agenda, HIV communities, researchers, and healthcare providers must enhance communication efforts to make information on HIV cure development more accessible, especially for those not connected to community organizations.

## CRediT authorship contribution statement

**Maaike A.J. Noorman:** Conceptualization, Data curation, Formal analysis, Investigation, Methodology, Project administration, Visualization, Writing – original draft, Resources. **John B.F. de Wit:** Conceptualization, Funding acquisition, Methodology, Supervision, Writing – review & editing. **Tamika A. Marcos:** Investigation, Project administration, Resources, Writing – review & editing. **Sarah E. Stutterheim:** Conceptualization, Funding acquisition, Supervision, Writing – review & editing. **Thijs Albers:** Investigation, Resources, Writing – review & editing. **Kai J. Jonas:** Conceptualization, Funding acquisition, Investigation, Supervision, Writing – review & editing. **Chantal den Daas:** Conceptualization, Funding acquisition, Methodology, Supervision, Writing – review & editing.

## Declaration of generative AI and AI-assisted technologies in the writing process

During the preparation of this work the authors used ChatGPT and Grammerly in order to enhance clarity and conciseness in writing. After using this, the authors reviewed and edited the content as needed and take full responsibility for the content of the publication**.**

## Funding

Aidsfonds funded this research under Grant P-53001.

## Declaration of competing interest

The authors declare the following financial interests/personal relationships which may be considered as potential competing interests: The authors report there are no direct competing interests to disclose. However, two of the authors (Sarah E. Stutterheim and Kai J. Jonas) received funding, unrelated to this topic from 10.13039/100005564Gilead, 10.13039/100030732MSD, and 10.13039/100010877ViiV Healthcare.

## Data Availability

Data will be made available on request.
