# Supplementary material for: Relatively high interest but limited active engagement in HIV cure research: Awareness, interest, and information-seeking among affected communities in the Netherlands
Source: J Virus Erad. 2024 Dec 9;10(4):100570. doi: 10.1016/j.jve.2024.100570 (PMC11697764; doi:10.1016/j.jve.2024.100570)
Supplement: Multimedia component 1 [file mmc1.docx]

| **Table S1 The adopted brief illness perception questionnaire (Brief IPQ) and principle component factor loadings** | | | | | | | | |  |
| --- | --- | --- | --- | --- | --- | --- | --- | --- | --- |
| **Construct** | **Original brief IPQ** | **Adapted version for people with HIV** | | | **Adapted version for key populations** | | | | |
|  |  |  | Factor Loading | |  | Factor Loading | | |  |
|  |  |  | Factor 1 | Factor 2 |  | Factor 1 | Factor 2 | Factor 3 |  |
| Consequence | How much does your illness affect your life? | To what extent does HIV influence your life? | **0.842** | 0.001 | How much does the chance that you might get HIV influence your life? | **0.832** | -0.018 | -0.095 |  |
| Personal control | How much control do you feel you have over your illness? | How much control do you think you have over HIV? | **0.487** | **0.418** | How much control do you think you have over whether or not you can get HIV? | 0.222 | **0.656** | -0.066 |  |
| Identity | How much do you experience symptoms from your illness? | To what extent do you experience symptoms from HIV? | **0.755** | 0.067 | To what extent do you think someone with HIV would experience symptoms related to HIV? | 0.027 | -0.042 | **0.977** |  |
| Treatment control | How much do you think your treatment can help your illness? | How much do you think ART can help with the treatment of HIV? | 0.110 | **0.692** | To what extent do you think HIV medication (ART) can help HIV treatment? | -0.046 | **0.761** | 0.152 |  |
| Concern | How concerned are you about your illness? | How concerned are you about your own HIV infection? | **0.760** | 0.205 | How concerned are you about HIV? | **0.873** | 0.053 | 0.012 |  |
| Emotions | How much does your illness affect you emotionally? (e.g., does it make you angry, scared, upset, or depressed?) | How much does HIV influence your mood (for example, does it make you angry, scared, upset or sad)? | **0.821** | 0.083 | How much does the possibility of you contracting HIV influence your mood (for example, does it make you angry, scared, upset or sad)? | **0.799** | -0.027 | 0.131 |  |
| Comprehensibility | How well do you feel you understand your illness? | How well do you feel you understand HIV? | 0.009 | **0.808** | How much knowledge do you feel you have about HIV? | -0.144 | **0.656** | -0.066 |  |

*Note*. Extraction Method: Principal component analysis. Rotation method: varimax with Kaiser normalization
All **bold** factor loadings are stable factor loadings (>0.400).
